# Clinical and molecular characteristics of two transaldolase-deficient patients

**DOI:** 10.1007/s00431-014-2261-2

**Published:** 2014-02-05

**Authors:** Anna Tylki-Szymanska, Mirjam M. C. Wamelink, Teresa J. Stradomska, Gajja S. Salomons, Joanna Taybert, Nel Dąbrowska-Leonik, Małgorzata Rurarz

**Affiliations:** 1 Department of Metabolic Diseases, The Children’s Memorial Health Institute, Al. Dzieci Polskich 20, 04-730 Warsaw, Poland; 2 Department of Clinical Chemistry, Neuroscience Campus Amsterdam, VU University Medical Centre, Amsterdam, The Netherlands; 3 Department of Biochemistry and Experimental Medicine, The Children’s Memorial Health Institute, Al. Dzieci Polskich 20, 04-730 Warsaw, Poland; 4 Department of Gastroenterology, Hepatology and Immunology, The Children’s Memorial Health Institute, Al. Dzieci Polskich 20, 04-730 Warsaw, Poland

**Keywords:** Transaldolase deficiency, Polyol concentration, Seven-carbon chain carbohydrates, Pentose phosphate pathway

## Abstract

Transaldolase (TALDO) deficiency is a rare metabolic disease in the pentose phosphate pathway, which manifests as a severe, early-onset multisystem disease. The body fluids of affected patients contain increased polyol concentrations and seven-carbon chain carbohydrates. We report the molecular and clinical findings in two recently diagnosed transaldolase-deficient children, both presented at birth. During infancy, they presented thin skin with a network of visible vessels, spider telangiectasias and multiple haemangiomas. Such unusual skin changes are characteristic of liver damage. Later, the patients developed rapidly progressive nodular liver fibrosis, tubulopathy and severe clotting disturbances. The clinical features of these patients were in line with previously studied patients with transaldolase deficiency. The diagnosis was established by detecting high concentrations of erythritol, ribitol, arabitol, sedoheptitol, perseitol, sedoheptulose and sedoheptulose-7-phosphate in the urine. Detection was made by gas chromatography and liquid chromatography-tandem mass spectrometry and then confirmed by molecular analysis of the TALDO gene. *Conclusion*: Transaldolase deficiency, a rare early-onset multisystem disease, should be considered by neonatologists, paediatricians, hepatologists and nephrologists in the differential diagnosis of patients presenting hepatosplenomegaly, thrombocytopenia, anaemia, bleeding diathesis, liver failure and tubulopathy.

## Introduction

Transaldolase (TALDO) deficiency (OMIM 606003) is an inborn error of the pentose phosphate pathway, which is a severe, early-onset multisystem disease. The body fluids of affected patients contain increased concentrations of polyol, heptulose, sedoheptulose, mannoheptulose and sedoheptulose-7P, mostly in the urine [8].

Patients present severe symptoms during the neonatal period, and in almost all cases, some signs were already noted prenatally. The leading symptoms in transaldolase-deficient patients are anaemia, bleeding problems with thrombocytopenia, hepatosplenomegaly, nodular progressive hepatic fibrosis and later on nephropathy. To date, 23 patients from 13 families have been described [1, 3, 6–9, 11]. The majority of patients have consanguineous parents.

**Fig. 1:**
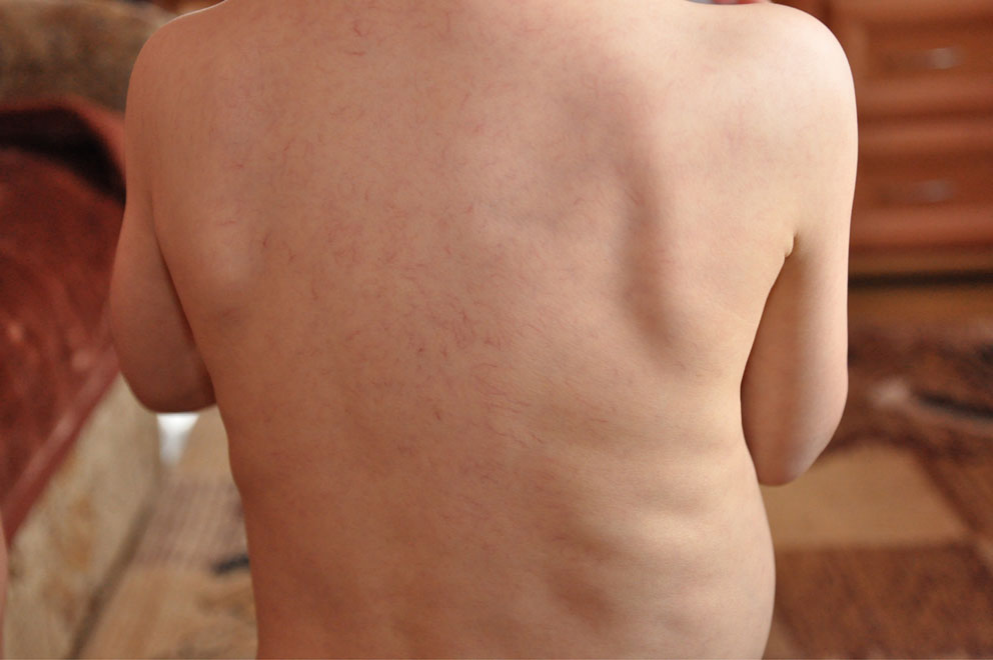
Skin changes characteristic of liver damage that immediately made us consider transaldolase deficiency

We present the clinical biochemical and molecular findings of two recently diagnosed transaldolase-deficient patients from two unrelated Polish families. Two boys, currently 2 and 3 years old, presented intrauterine growth retardation (IUGR), ascites and oligoamnios. Postnatally, they presented anaemia, bleeding diathesis, hepatosplenomegaly and liver involvement. In contrast with their physical development, psychomotor development was normal. Both presented a characteristic of thin skin with a network of visible vessels, spider telangiectasias, and multiple haemangiomas. These skin changes, characteristic of liver damage, immediately made us consider transaldolase deficiency (Fig 1).

## Materials and methods

### Patient 1 (KL)

A boy, the first born child of unrelated parents, was noted to have oligoamnios as well as foetal growth retardation. He was delivered by caesarean section at 41 weeks. At birth, the boy presented low body weight (2,150 g), hepatosplenomegaly, anaemia and clotting diathesis.

At the age of 1 month, he was hospitalised because of hepatosplenomegaly, anaemia and failure to thrive (low body mass). At that time, thin skin with a network of visible vessels, spider angiomas and multiple *haemangiomas* were noted. Additional investigations revealed blood clotting disturbances, prolonged prothrombin time (PT), activated partial thromboplastin time (APTT), deficiency in factors XI XII, elevated transaminases, hypoproteinemia, anaemia (Hb 7,3, E 2,97), thrombocytopenia and nodular changes in the liver. These features suggested TALDO deficiency, and therefore, sugars and polyols were determined. Elevated excretion of erythritol, ribitol, arabitol, sedoheptitol, perseitol, sedoheptulose and sedoheptulose-7P in the urine was detected by gas chromatography (GC) and liquid chromatography-tandem mass spectrometry (LC-MS/MS; Table 1), which is indicative of TALDO deficiency [5, 8, 9]. A homozygous deletion (c.462-174_981 + 53del) in the *TALDO* gene was expected because of the fact that exon 5 could not be amplified by PCR in contrast to all other exons. The deletion was confirmed by long-range PCR followed by sequencing of the region of exons 4 to 8. This revealed a deletion of approximately 1.3 kb comprising the 3′-end of intron 4 and the 5′-end of intron 7.

**Table 1:** Urinary concentrations of polyols and seven-carbon sugars of the two patients compared with reference values

Metabolite (urine spot^a^)	Patient 1 (KL, 34 weeks)	Reference values	Patient 2 (SA, 1.4 year)	Reference values
mmol/mol creatinine				
Erythritol^b^	*687*	89–158	*360*	76–192
Arabitol^b^	*588*	51–99	*354*	52–88
Ribitol^b^	*432*	10–17	*209*	9–24
Sedoheptitol^c^	*13*	<1	*↑* ^*b*^	<1
Perseitol^c^	*67*	<1	nd	<1
Sedoheptulose^c^	*≥2,500*	<9	*↑* ^*b*^	<9
Mannoheptulose^c^	Disturbed	<3	nd	<3
Sedoheptulose-7P^c^	*1.29*	<0.12	*3.71*	<0.12

Values in italics are elevated compared with age-related reference values. Disturbed denotes that mannoheptulose is not quantitative due to very high excretion of sedoheptulose

*nd* not done

^a^Metabolites were measured in urine spotted on filter paper and afterwards extracted with water. Some sugars are not stable on filter paper; therefore, the actual concentrations might have been higher

^b^LC/MS-MS method [10]

Currently, the boy is 3 years old. He presents anaemia, bleeding diathesis, thrombocytopenia, elevated transaminases, signs of tubulopathy, renal tubular dysfunction (proteinuria, aminoaciduria, uricuria and hypercalciuria, renal acidosis) and an increased number of nodular changes in his liver.

### Patient 2 (SA)

He is the first born of young non-consanguineous parents. During pregnancy, growth retardation of the foetus was noted; ultrasonography revealed fluid in the pericardium and abdominal cavity. Labour at 37 weeks was uncomplicated; his birth weight was 2,410 g. During the neonatal period, he presented an enlarged spleen and liver, cryptorchismus, skin changes in the form of visibly dilated vessels, spider angiomas and cavernous haemangiomas*.* Laboratory investigations showed anaemia, thrombocytopenia, lymphopenia, blood clotting disturbances, PT, APTT, deficiency in atythrombin III, elevated bilirubin and gamma-glutamyltransferase, mildly elevated transaminases and elevated alpha-fetoprotein. At the age of 6 months, renal calculus was found; few small calculi in both kidneys were detected by ultrasound. Magnetic resonance of the liver revealed multiple fibrotic nodular changes and malformation of the left branch of the hepatic portal vein.

At the age of 15 months, urinary sugars and polyols were measured using GC, and the profile suggested transaldolase deficiency.

Molecular analysis revealed two mutations, (c.575G > A; p.(Arg192His) and (c.462-174_981 + 53del), in the *TALDO* gene. Both parents are carriers of one of the described mutations, confirming compound heterozygosity in the child. The c.575G > A mutation had been found before in patients affected with TALDO deficiency [2]. The second allele initially remained unnoticed, but, since tests in our laboratory had previously detected homozygous deletion in the DNA of patient 1 (this report), we decided to investigate, by means of long-range PCR, whether this deletion might also be present in heterozygous form in the DNA of patient 2. This confirmed the diagnosis.

Currently, the patient is 2 years old and has severe clotting disturbances as well as anaemia, thrombocytopenia and pancytopenia. He also developed tubulopathy and numerous calculi in both kidneys. The progress of liver damage and renal involvement is dynamic and suggests a poor outcome.

## Discussion

TALDO deficiency results in a severe metabolic disturbance which affects foetal development and produces characteristic signs such as placental overgrowth, amniotic and foetal body fluid imbalance, and intrauterine growth retardation [1, 3, 6–8, 11]

Prenatally, our two patients with TALDO deficiency already presented intrauterine growth retardation, ascites and oligoamnios. Postnatally, they presented anaemia, bleeding diathesis, hepatosplenomegaly, liver involvement and overall poor physical development. Their mental development is normal.

The clinical features were characteristic of TALDO deficiency, and diagnosis was confirmed via biochemical and molecular methods. In the second case, we first detected, by way of Sanger sequencing of PCR products, only one heterozygous missense mutation. Heterozygous deletion could only be detected because of a detection in another Polish patient (i.e. patient 1 in this report)—a homozygous deletion that was confirmed by long-range PCR. Usually, heterozygous deletions are missed since most laboratories use direct DNA sequence analysis of PCR products, and this technique cannot detect copy number variations (e.g. heterozygous deletions of exons or duplications). This means that further work is needed if there is a strong suspicion based on clinical characteristics or biochemical basis. Also, if none or only one of the mutations is detected, then further work is needed. This could include mRNA analysis and/or specific tests for the presence of genomic deletions, such as multiplex probe amplification tests or, if available, long-range PCR.

**Fig 2:**
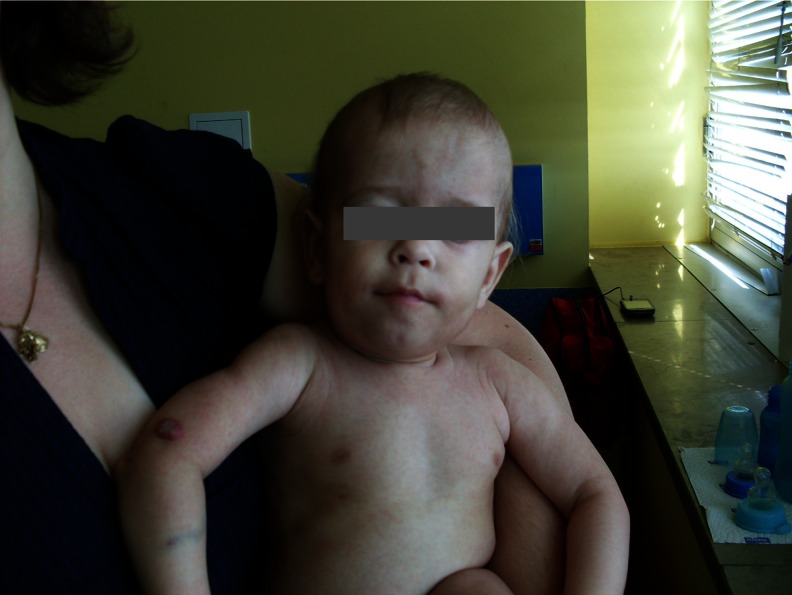
Skin haemangioma in patient with transaldolase deficiency

Both patients presented a characteristic of thin skin with a network of visible vessels, spider angiomas and multiple haemangiomas (Fig. 1). Curtis laxa or dysmorfism described by other authors was not present in our patients [3, 7]. In patient 2, liver vessel malformation and skin haemangiomas (*blood vessel malformations*; Fig. 2) were present. These skin changes were observed in other patients [3, 6], and it seems to be characteristic of patients with transaldolase deficiency. Spider angiomas and blood vessel dilatation are related to significant damage of the liver. Spider angiomas are present in adult patients when their liver cannot detoxify oestrogen from the blood, resulting in high levels of oestrogen [4]. In cases of TALDO deficiency, liver damage begins at the foetal stage, resulting in clotting disturbances, elevated transaminases, hypoalbuminemia and characteristic changes of skin vessels in the newborn. The latter suggests a toxic impact of accumulated sugars and polyols on the liver, which starts on early foetal life. Patients with TALDO deficiency are already born with advanced disease primarily affecting not only the liver but also the kidneys, vessels, and other systems. However, in the differential diagnosis, syndromes with liver failure and tubulopathy like tyrosinemia type I and Fanconi–Bickel syndrome could be considered.

The development of tubulopathy and, in the case of the second patient, nephrolithiasis is probably due to the toxic effect of sugars, sugar phosphates and polyols on the proximal tubules, leading to kidney failure [5].

Haemangiomas present in our and other patients may be secondary to the disturbed placental formation and function [2]. Placental abnormalities in TALDO deficiency have been described by other authors [1, 3, 6, 7, 11]. Symptomatic treatment can be proposed. Liver transplantation in the early stage of the disease could perhaps be useful.
